# Prognostic value of albumin-based malnutritional indices on short-term outcome in acute ischemic stroke patients undergoing reperfusion therapy

**DOI:** 10.3389/fnut.2025.1659446

**Published:** 2025-08-04

**Authors:** Junle Zhao, Mingjie Chen, Junning Mo, Yutong Zhong, Jiamin Qiu, Yanjiao Qiu, Youjia Li, Xiaoyan Han

**Affiliations:** ^1^Department of Neurology, First People’s Hospital of Zhaoqing, Zhaoqing, China; ^2^Clinical Research Center, First People’s Hospital of Zhaoqing, Zhaoqing, China

**Keywords:** malnutrition, albumin, acute ischemic stroke, reperfusion therapy, prognosis, the controlling nutritional status score, the prognostic nutritional index score, neutrophil percentage-to-albumin ratio

## Abstract

**Background:**

The malnutrition and inflammatory status are dependently associated with an increased risk of poor prognosis in patients with acute ischemic stroke (AIS). However, the evidence surrounding the prognostic significance of albumin-based malnutritional indices in AIS patients receiving reperfusion therapy remains insufficient. We aimed to explore prognostic value of the controlling nutritional status score (CONUT), prognostic nutritional index score (PNI), and neutrophil percentage-to-albumin ratio (NPAR) on short-term outcome of AIS patients undergoing reperfusion therapy.

**Methods:**

A total of 612 AIS patients were enrolled. The association of the malnutritional indices and 3-months poor prognosis was accessed by multivariable logistics regression model. We further performed a logistic regression model with restricted cubic splines to examine the potential nonlinear correlations between the malnutritional indices and short-term poor prognosis. Subgroup analysis and reclassification indexes were also conducted to enhance the robustness of the findings. Additionally, mediation analyses were performed to examined the potential mediating effects of early neurological deterioration (END) presence on the associations of malnutrition with 3-months unfavorable outcomes.

**Results:**

256 patients developed poor prognosis at 3-month follow-up. Malnutrition was associated with the risk of 3-month poor functional outcome for CONUT (per 1-point increased, adjusted OR 1.59, 95%CI 1.40–1.82), for PNI (per 1-point increased, adjusted OR 0.86, 95%CI 0.82–0.90), and the NPAR (per 1-SD increased, adjusted OR 23.97, 95%CI 7.15–85.72). The PNI exhibited nonlinear association with the 3-month poor prognosis. These three indices in addition to other clinical risk factors improved the model discrimination. Compared to the NPAR, the net reclassification improvement of PNI (0.579, 95%CI 0.418–0.739) and CONUT (0.631, 95%CI 0.470–0.792) were higher in predicting short-term poor prognosis. The findings were further supported by subgroup analyses, and END had no significant mediated effects on the associations of malnutrition and 3-month unfavorable outcome.

**Conclusion:**

Albumin-based malnutritional indices are reliable and feasible prognostic indicators in AIS patients, predicting short-term outcome independent of the type of reperfusion treatment. The applicability of these objective malnutritional indices may improve risk stratification and guide nutritional interventions at clinical setting in this vulnerable ischemic stroke population.

## 1 Introduction

Acute ischemic stroke (AIS) is triggered by abrupt interruption of cerebral blood flow causing devastating brain damage and severe neurological deficits (1, 2), and it continues to rank as a primary cause of death and persistent disability globally (3). Rapid blood flow reperfusion therapy using intravenous thrombolysis (IVT) or/and endovascular thrombectomy (EVT) has greatly advanced the treatment of AIS. However, approximately 1 of 4 patients have poor outcome despite technically successful intervention (4). Therefore, strategies for the accurate prediction of neurological outcomes in at-risk individuals hold substantial clinical significance, potentially presenting an alternative framework for patient monitoring and improving post-reperfusion therapy outcomes.

The incidence of premorbid malnutrition risk was around 34% among AIS patients at admission (5, 6), and has been consistently associated with unfavorable outcome in both short- and long-term period (7–9). Beyond the cerebral deficits induced by the ischemic insult, inflammation and oxidative stress exert a pivotal role in the cascade of cerebral ischemic reperfusion injury (10). Particularly among patients undergoing IVT or EVT, the pre-existing nutritional status deteriorates rapidly after stroke onset due to stress associated with severe catabolism (11), and the interaction between post-stroke inflammatory injury and malnutrition is likely to be more pronounced (12, 13). Thus, early prognostic immuno-nutritional assessment is of critical importance among AIS patients, facilitating timely risk stratification and targeted nutritional intervention.

However, malnutrition in AIS patients is frequently overlooked by clinicians. The challenges in the assessment of nutritional status among AIS patients may account for this scenario. Aphasia, confusion and immobile symptoms of stroke patient may pose a considerable challenge to conventional nutritional assessments, such as body mass index (BMI) and nutritional assessment tool depending on the dietary history (14). Notably, the objective nutritional markers forecasting neurological outcomes during the early stage of stroke have attracted attention in recent years. The controlling nutritional status score (CONUT), the prognostic nutritional index score (PNI), and the neutrophil percentage-to-albumin ratio (NPAR) which can be easy to calculate using blood-based parameters and could be feasible to reflect balance of immune-nutritional status in clinical practice (15, 16). Previous studies on the prognostic significance of PNI and CONUT in AIS patients have primarily focused on individuals who did not undergo reperfusion therapy (17, 18), or patients treated with IVT or EVT alone (5, 7, 15, 19). As an emerging biomarker for assessing immune and systemic nutritional conditions, NPAR is increasingly being used in studies on disease risk and prognosis. Recently a few studies have indicated that NPAR is associated with stroke recurrence at 3-month follow-up (20), and early neurological improvement in AIS patients after IVT (21). Collectively, these findings suggested a potential association between albumin-based indicators integrating inflammation and nutritional status and the prognosis of AIS patients undergoing reperfusion therapy.

However, which composite indicator is more indicative of short-term adverse outcome risk in AIS patients undergoing reperfusion therapy (IVT/EVT alone or combined strategies) remains unclear and merits further exploration, particularly within the context of ischemic stroke treatment that prioritizes rapid reperfusion therapy. To address these gaps, we employed three objective nutritional indices to predict 3-month outcomes in AIS patients receiving reperfusion therapy and to ascertain which nutritional assessment tool is more suitable to early identify the risk in such stroke population.

## 2 Materials and methods

### 2.1 Study population

This was a STROBE-compliant, retrospective study conducted at First People’s Hospital of Zhaoqing in China, including consecutive AIS patients admitted between January 2019 and January 2024. Patients were included if they (a) age ≥18 years old, (b) times since ischemic stroke onset within 6 h, (c) who were diagnosed with ischemic stroke confirmed by brain computed tomography (CT) or magnetic resonance imaging (MRI); (d) treatment with IVT/EVT alone, or EVT in combination with IVT (bridging strategy) after admission, and (e) had 3-month post-discharge follow-up record. Patients were excluded if they met any of the following criteria: (a) incomplete laboratory data within 24 h of admission; (b) a history of systemic inflammatory diseases, malignant tumors, or hematological diseases; (c) a history of severe hepatic, renal, or cardiac dysfunction.

### 2.2 Ethics statement

This retrospective observational study obtained ethical approval from the Ethics Review Committee of the First People’s Hospital of Zhaoqing (approval number: B2022-11-07). It was conducted retrospectively using clinical records and strictly adhered to the Declaration of Helsinki for human research. After collecting clinical information, patient identifiers were de-identified to ensure neither direct nor indirect patient identification. This study was approved by the hospital’s Ethics Committee, which waived the requirement for signed informed consent.

### 2.3 Demographic and clinical data

Detail demographic data including age, sex, history of hypertension, diabetes mellitus (DM), coronary atherosclerotic heart disease (CHD), prior ischemic stroke, and smoking status, was retrieved from electronic medical records at admission. All laboratory tests (complete blood count, liver function tests, renal function tests, and lipid profiles) were defined as the initial test results obtained within 24 h of admission. The severity of stroke was assessed by trained neurologists utilizing the National Institutes of Health Stroke Scale (NIHSS) (22). Stroke etiology were classified according to the trial of ORG 10172 in Acute Stroke Treatment (TOAST) categorization (23). Early neurological deterioration (END) was defined as an increase of ≥4 points in the NIHSS score within 72 h of admission (24).

### 2.4 Malnutrition screening tools

The CONUT score was calculated using serum albumin concentration, total peripheral lymphocyte count, and total cholesterol concentration (25). The PNI was computed using the formula: [5 × lymphocyte count (10^9^/L) + serum albumin (g/L)] (26, 27). Table 1 indicated the scoring systems of CONUT and PNI as recommended by original papers. According to the prior study, NPAR was defined as the percentage of neutrophils divided by the albumin levels (16).

**Table 1:** Details of the CONUT and PNI scoring systems.

Nutrition scores	Risk of malnutrition			
	Absent	Mild	Moderate	Severe
CONUT, points	0–1	2–4	5–8	9–12
Albumin, g/l	35	30–34	25–29	<25
Score	0	2	4	6
Total cholesterol, mg/dl	180	140–179	100–139	<100
Score	0	1	2	3
Lymphocyte count, × 10^9^/l	1.60	1.20–1.59	0.80–1.19	<0.80
Score	0	1	2	3
PNI, points	>38		35–38	<35

CONUT, controlling nutritional status score; PNI, prognostic nutritional index.

### 2.5 Definition of clinical outcome

The patient’s short-term functional recovery was assessed by a neurologist or trained specialist nurse using the modified Rankin Scale (mRS) via face-to-face or telephone interview at the 3-month follow-up. The poor clinical outcome was defined as an indicator of functional independence with a mRS ranges from 3 to 6 (28).

### 2.6 Statistical analysis

Continuous variables were expressed as the mean with SD or the median with interquartile range (interquartile range [IQR], 25–75%), as appropriate. The normality distribution of each parameter was tested by the Shapiro–Wilk test. According to the normality distribution test, the Student t-test or the Mann–Whitney-U test was used to compare the two groups involving continuous variables. Categorical data was presented as numbers and (percentages, %), and the Chi-squared test or Fisher exact test, as appropriate, was used to compare the two groups. The CONUT score was analyzed as a continuous variable and categorized into three groups (absent, mild, moderate to severe), as there were too few patients in the severe category for detailed analysis. The PNI and NPAR score were evaluated continuously and categorically in tertile. The independent relationships between the malnutrition indices and poor outcome of AIS patients were investigated by multivariable binary logistic regression analyses. Model 1 was adjusted for age and sex. Model 2 was adjusted for age, baseline laboratory parameter (white blood cell, triglyceride, fasting blood sugar), NIHSS at admission, and adding with vascular risk factors at admission (smoking status, and history of coronary atherosclerotic heart disease and hypertension). We selected the variables with P<0.05 threshold associated with poor clinical outcome in univariate analysis into model 3. The model selection was conducted using a stepwise selection procedure, excluding hematologic indices that were included in the calculations of the CONUT, PNI, and NPAR scores. The clinically relevant variables (age, history of ischemic stroke, history of diabetes mellitus, systolic blood pressure at baseline, stroke etiology, the level of carotid atherosclerosis, peripheral white blood cell count, hemoglobin, triglyceride, fasting blood sugar, international normalized ratio (INR), NIHSS at admission, type of reperfusion therapy) were considered independent covariates in model 3. Furthermore, we used receiver operator characteristic (ROC) curve analysis to verify and compare the predictive validity of the malnutritional indices for clinical outcomes. To estimate the improvement of predictive performance after adding malnutritional indices into different model 3, we employed the net reclassification index (NRI) and integrated discrimination improvement (IDI) (29). Subsequently, decision curve analysis (DCA) was also used to compare the clinical benefits and performance improvements of different models with or without the inclusion of malnutritional indices (30).

We further performed a logistic regression model with restricted cubic splines (RCS) adjusted for the same covariates included in model 3 to examine the potential nonlinear correlations between the malnutritional indices and short-term poor prognosis after AIS onset. We applied RCS with 5 knots (at fifth, 27.5th, 50th, 72.5th, 95th percentiles) to balance best fit and overfitting (31).

Subgroup analyses and interaction tests were conducted to evaluate differences across age subgroups, stroke subtypes, reperfusion therapy types, stroke severities, and whether it was the first-ever ischemic stroke. The association between the three malnutritional indices and poor clinical outcomes in each subgroup was assessed using logistic regression models adjusted for the covariates from model 3. Additionally, interaction tests were performed for the aforementioned stratification factors.

Given that END serves as an established risk factor for 3-months poor prognosis in AIS (18), we first examined the potential mediating effects of END presence on the associations of malnutrition (shown as malnutritional index) with 3-months poor prognosis risk were estimated by parallel mediation. Analyses were performed by R software (version 4.0.5, R Foundation, Vienna, Austria), and a *p* value < 0.05 was considered as statistical significance.

## 3 Results

**Figure 1:**
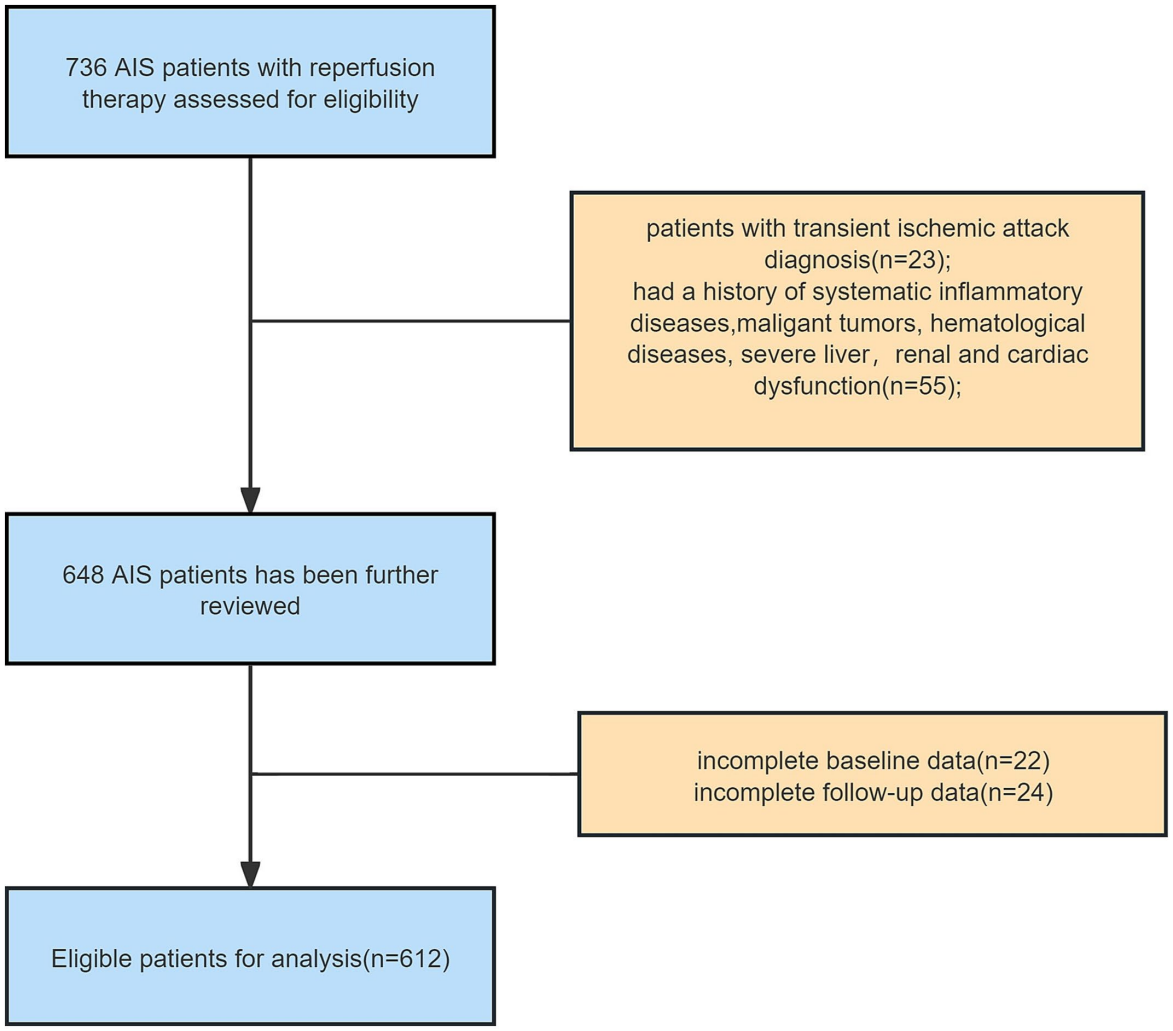
The flow chart of the current study. AIS, acute ischemic stroke.

Of the 736 participants consecutively screened at baseline, 55 (7.5%) individuals were excluded for failure to meet baseline eligibility criteria, 23 (3.1%) for transient ischemic attack diagnosis, and 46 (6.3%) due to loss to follow-up or unavailable laboratory test data. Overall, 612 (83.2%) participants were eligible for the final analysis (Figure 1).

### 3.1 Clinical characteristics of the patients enrolled in the study

The mean age of the enrolled patients was 66 (57, 73) years, and 434 (70.9%) of them were male. 256 (41.8%) patients undergoing IVT or/and EVT were classified into the poor prognosis group (mRS score range from 3 to 6) at three-months follow-up. Detailed baseline clinical characteristics are shown in Table 2. Briefly, patients in the poor prognosis group were older, had a higher prevalence of diabetes and previous ischemic stroke history, more often have EVT alone or combined with IVT treatment, and more severe NIHSS score at admission (all *p* < 0.05). The poor prognosis group exhibited significantly higher levels of white blood cell count, neutrophil count, monocyte count, compared with the good prognosis group. Conversely, the poor prognosis group demonstrated significantly lower levels of lymphocyte, hemoglobin, albumin, and total cholesterol compared with the good prognosis group (*p* < 0.05). Patients with poor prognosis were more likely to present with poorer nutritional status at admission: lower PNI score (median [IQR], 43.10 [39.65, 48.07] points vs. 47.88 [44.59, 51.52] points, *p* < 0.001), higher NPAR score (mean [SD], 2.03 ± 0.41 points vs.1.75 ± 0.41 points, *p* < 0.001), and higher CONUT score (median [IQR], 3.00 [1.00, 4.00] points vs. 1.00 [0.00, 2.00] points, *p* < 0.001).

**Table 2:** Baseline characteristics at admission according to AIS patients receiving reperfusion therapy with and without poor neurological functional prognosis at three-month follow-up.

Variables	Overall (*n* = 612)	Good prognosis (*n* = 356)	Poor prognosis (*n* = 256)	*p*-value
Sex, Male *n* (%)	434 (70.9)	253 (71.1)	181 (70.7)	0.994
Age, Median (IQR)	66.00 [57.00, 73.00]	65.00 [56.00, 71.00]	69.00 [61.00, 76.00]	<0.001^*^
DM, *n* (%)	110 (18.0)	51 (14.3)	59 (23.0)	0.008^*^
HTN, *n* (%)	301 (49.2)	169 (47.5)	132 (51.6)	0.359
IS, *n* (%)	113 (18.5)	54 (15.2)	59 (23.0)	0.018^*^
CHD, *n* (%)	54 (8.8)	30 (8.4)	24 (9.4)	0.792
Current smoking, *n* (%)	229 (37.4)	131 (36.8)	98 (38.3)	0.772
Early neurology deterioration, *n* (%)	65 (10.6)	10 (2.8)	55 (21.5)	<0.001^*^
Reperfusion therapy				<0.001^*^
Thrombolysis	404 (66.0)	271 (76.1)	133 (52.0)	
Endovascular treatment	145 (23.7)	60 (16.9)	85 (33.2)	
Both	63 (10.3)	25 (7.0)	38 (14.8)	
NIHSS at admission, Median (IQR)	7.00 [3.00, 14.00]	4.00 [2.00, 8.25]	12.00 [7.00, 19.00]	<0.001^*^
mRS before admission, Median (IQR)	0.00 [0.00, 0.00]	0.00 [0.00, 0.00]	0.00 [0.00, 0.00]	0.019^*^
Atrial fibrillation, *n* (%)	43 (7.0)	19 (5.3)	24 (9.4)	0.077
Carotid atherosclerosis, *n* (%)				<0.001^*^
Non	151 (24.7)	113 (31.7)	38 (14.8)	
Carotid plaque	201 (32.8)	124 (34.8)	77 (30.1)	
Carotid severe stenosis	260 (42.5)	119 (33.4)	141 (55.1)	
TOAST, *n* (%)				<0.001^*^
LAA	400 (65.4)	202 (56.7)	198 (77.3)	
CE	44 (7.2)	27 (7.6)	17 (6.6)	
SAA	130 (21.2)	101 (28.4)	29 (11.3)	
others	38 (6.2)	26 (7.3)	12 (4.7)	
SBP, Mean ± SD, mmHg	149.24 (23.06)	147.29 (23.84)	151.95 (21.68)	0.014^*^
WBC, Median (IQR), ×10^9^/l	8.68 [7.03, 10.81]	8.18 [6.84, 10.37]	9.38 [7.40, 11.60]	<0.001^*^
Nec, Median (IQR), ×10^9^/l	5.84 [4.36, 8.30]	5.28 [4.04, 7.32]	6.72 [4.90, 9.18]	<0.001^*^
LYM, Median (IQR), ×10^9^/l	1.63 [1.13, 2.32]	1.91 [1.34, 2.46]	1.41 [0.97, 2.07]	<0.001^*^
RBC, Median (IQR), ×10^12^/l	4.70 [4.31, 5.05]	4.71 [4.32, 5.06]	4.66 [4.29, 5.04]	0.413
Hemoglobin, (median [IQR])	139.00 [127.00, 149.00]	140.00 [129.00, 151.00]	136.00 [123.75, 147.00]	0.003^*^
ALT, Median (IQR), u/l	16.00 [12.00, 22.00]	17.00 [13.00, 23.00]	16.00 [12.00, 21.00]	0.035^*^
ALB, Median (IQR), g/l	37.40 [34.80, 39.90]	38.50 [36.30, 40.30]	35.30 [34.10, 38.32]	<0.001^*^
TC, Median (IQR), mmol/l	4.68 [3.97, 5.39]	4.79 [4.15, 5.50]	4.50 [3.79, 5.30]	0.005^*^
Triglyceride, (median [IQR])	1.18 [0.83, 1.80]	1.29 [0.87, 1.94]	1.08 [0.80, 1.61]	0.001^*^
Scr, Median (IQR), μmol/l	80.45 [67.20, 96.40]	80.10 [66.97, 96.20]	80.85 [68.33, 96.75]	0.647
FBS, Median (IQR), mmol/l	5.92 [5.11, 7.35]	5.59 [5.00, 6.56]	6.56 [5.42, 8.77]	<0.001^*^
HbA1c, Median (IQR), mmol/l	6.00 [5.66, 6.64]	6.00 [5.60, 6.60]	6.09 [5.70, 6.80]	0.117
INR (median [IQR])	0.98 [0.94, 1.04]	0.97 [0.93, 1.04]	1.00 [0.95, 1.06]	<0.001^*^
Fib, Median (IQR), g/l	3.11 [2.68, 3.66]	3.12 [2.68, 3.73]	3.10 [2.66, 3.64]	0.46
NPAR (mean (SD))	1.87 (0.43)	1.75 (0.39)	2.03 (0.41)	<0.001^*^
CONUT, Median (IQR)	2.00 [1.00, 3.00]	1.00 [0.00, 2.00]	3.00 [1.00, 4.00]	<0.001^*^
PNI (median [IQR])	46.40 [42.10, 50.11]	47.88 [44.59, 51.52]	43.10 [39.65, 48.07]	<0.001^*^
CONUT scoring system, *n* (%)				<0.001^*^
Absent	263 (43.0)	198 (55.6)	65 (25.4)	
Mild	273 (44.6)	139 (39.0)	134 (52.3)	
Moderate-severe	76 (12.4)	19 (5.3)	57 (22.3)	

AIS, acute ischemic storke; IQR, interquartile ranges; SD, standard deviation; DM, diabetes mellitus; HTN, hypertension; IS, ischemic stroke; CHD, coronary atherosclerotic heart disease; NIHSS, National Institute of Health Stroke Scale; mRS, modified Rankin score; TOAST, the trial of ORG 10172 in Acute Stroke Treatment; LAA, large-artery atherosclerosis; CE, cardioembolism; SAA, small-vessel occlusion; Others: include SOE, stroke of other determined etiology and SUE, stroke of undetermined etiology; SBP, systolic blood pressure; DBP, diastolic blood pressure; WBC, white blood cell; Nec, neutrophil; LYM, lymphocyte; Mo, monocyte; LYM, lymphocyte; RBC, red blood cell; ALT, alanine transaminase; ALB, albumin; TC, total cholesterol; Scr, serum creatinine; FBS, fasting blood sugar; HbA1c, glycated hemoglobin; PT, Prothrombin time; INR, international normalized ratio; Fib, Fibrinogen; NPAR, neutrophil percentage-to-albumin ratio; MAR, monocyte-to-albumin ratio; CONUT, controlling nutritional status score; PNI, prognostic nutritional index. Continuous variables are presented as mean±SD or median (interquartile range [IQR], 25–75%), with normality assessed via Shapiro–Wilk test. Between-group comparisons for normally distributed continuous variables used Student’s t-test, and Mann–Whitney U test for non-normal distributions. Categorical data are expressed as *n* (%), with Chi-square test or Fisher’s exact test applied as appropriate. Statistical significance was set at *p* < 0.05, and *p < 0.05 denotes significant differences between groups. Bold font values indicate statistically significant differences.

### 3.2 Association of malnutrition indices and 3-months poor outcome in AIS

On multivariable analysis, the three malnutritional indices were independently associated with three-month poor prognosis in AIS patients undergoing reperfusion therapy, regardless of whether the malnutritional indices were treated as continuous or categorical variables (Table 3). Compared with “absent risk” group, the baseline “mild risk” and “moderate–severe risk” groups were associated with poor prognosis at 3-months follow-up, as assessed by the CONUT (Model 3, mild risk: OR 2.99, 95%CI 1.85–4.89, *p* < 0.001; moderate - severe risk: OR 14.07, 95%CI 6.62–31.10, *p* < 0.001; P for trend: OR 2.06, 95%CI (1.56–2.74), *p* < 0.001). After categorizing PNI and NPAR into tertiles, we observed that individuals in the higher tertile groups of PNI score (Model 3, tertile2 risk: OR 0.25, 95%CI 0.15–0.41; tertile3 risk: OR 0.18, 95%CI 0.10–0.32; P for trend: OR 0.40, 95%CI (0.30–0.52); all *p* < 0.001), and the lower tertile groups of NPAR score (tertile2 risk: OR 1.95, 95%CI 1.06–3.62, *p* = 0.033; tertile3 risk: OR 4.02, 95%CI 1.75–9.44, *p* = 0.001; P for trend: OR 1.96, 95%CI (0.30–0.52), *p* < 0.001) were more likely to have a decreased risk of poor outcome in multivariable analysis adjusted covariates of model 3. In model 3, the significant association of the three malnutritional indices persisted when analyzed as continuous variables (all *p* < 0.001).

**Table 3:** Multivariable logistic regression analyses of the three malnutritional indices at admission to predict poor clinical outcome at 3 months after reperfusion therapy.

Index	Model 1^†^	*p*	Model 2^‡^	*p*	Model3 ^§^	*p*
	Adjusted OR (95%CI)		Adjusted OR (95%CI)		Adjusted OR (95%CI)	
PNI categories						
Tertile1 (<43.60)	1.0 [Reference]		1.0 [Reference]		1.0 [Reference]	
Tertile2 (43.60–48.95)	0.27 (0.17–0.41)	**<0.001**	0.31 (0.19–0.50)	**<0.001**	0.25 (0.15–0.41)	**<0.001**
Tertile3 (>48.95)	0.20 (0.13–0.32)	**<0.001**	0.23 (0.14–0.39)	**<0.001**	0.18 (0.10–0.32)	**<0.001**
*P* for trend	0.41 (0.32–0.53)	**<0.001**	0.45 (0.34–0.59)	**<0.001**	0.40 (0.30–0.52)	**<0.001**
PNI per 1-point increase	0.87 (0.83–0.90)	**<0.001**	0.88 (0.84–0.91)	**<0.001**	0.86 (0.82–0.90)	**<0.001**
CONUT categories						
Absent	1.0 [Reference]		1.0 [Reference]		1.0 [Reference]	
Mild	3.50 (2.31–5.40)	**<0.001**	2.67 (1.68–4.29)	**<0.001**	2.99 (1.85–4.89)	**<0.001**
Moderate-severe	12.32 (6.73–23.28)	**<0.001**	9.74 (4.87–20.05)	**<0.001**	14.07 (6.62–31.10)	**<0.001**
*P* for trend	2.15 (1.71–2.72)	**<0.001**	1.79 (1.38–2.33)	**<0.001**	2.06 (1.56–2.74)	**<0.001**
CONUT per 1-point increase	1.59 (1.43–1.78)	**<0.001**	1.49 (1.32–1.69)	**<0.001**	1.59 (1.40–1.82)	**<0.001**
NPAR categories						
Tertile1 (<1.69)	1.0 [Reference]		1.0 [Reference]		1.0 [Reference]	
Tertile2 (1.69–2.07)	2.09 (1.31–3.36)	**0.002**	2.11 (1.17–3.86)	**0.014**	1.95 (1.06–3.62)	**0.033**
Tertile3 (>2.07)	4.28 (2.72–6.86)	**<0.001**	4.15 (1.85–9.54)	**0.001**	4.02 (1.75–9.44)	**0.001**
*P* for trend	2.28 (1.81–2.89)	**<0.001**	1.86 (1.40–2.48)	**<0.001**	1.96 (0.30–0.52)	**<0.001**
NPAR per 1-SD increase	29.23 (11.85–75.90)	**<0.001**	15.44 (5.02–50.08)	**<0.001**	23.97 (7.15–85.72)	**<0.001**

†Model 1, adjusted for age, sex.

‡Model 2, adjusted for age, history of coronary atherosclerotic heart disease, history of hypertension, white blood cell, triglyceride, fasting blood sugar, NIHSS at admission.

§Model 3, adjusted for age, history of ischemic stroke, history of diabetes mellitus, systolic blood pressure, stroke etiology, the level of carotid atherosclerosis, white blood cell, Hemoglobin, triglyceride, fasting blood sugar, international normalized ratio, NIHSS at admission, treating-type of reperfusion therapy.

OR, odds ratio; CI, confidence interval; SD, standard deviation; PNI, prognostic nutritional index. CONUT, controlling nutritional status score; NPAR, neutrophil percentage-to-albumin ratio. Bold font values indicate statistically significant differences.

**Figure 2:**
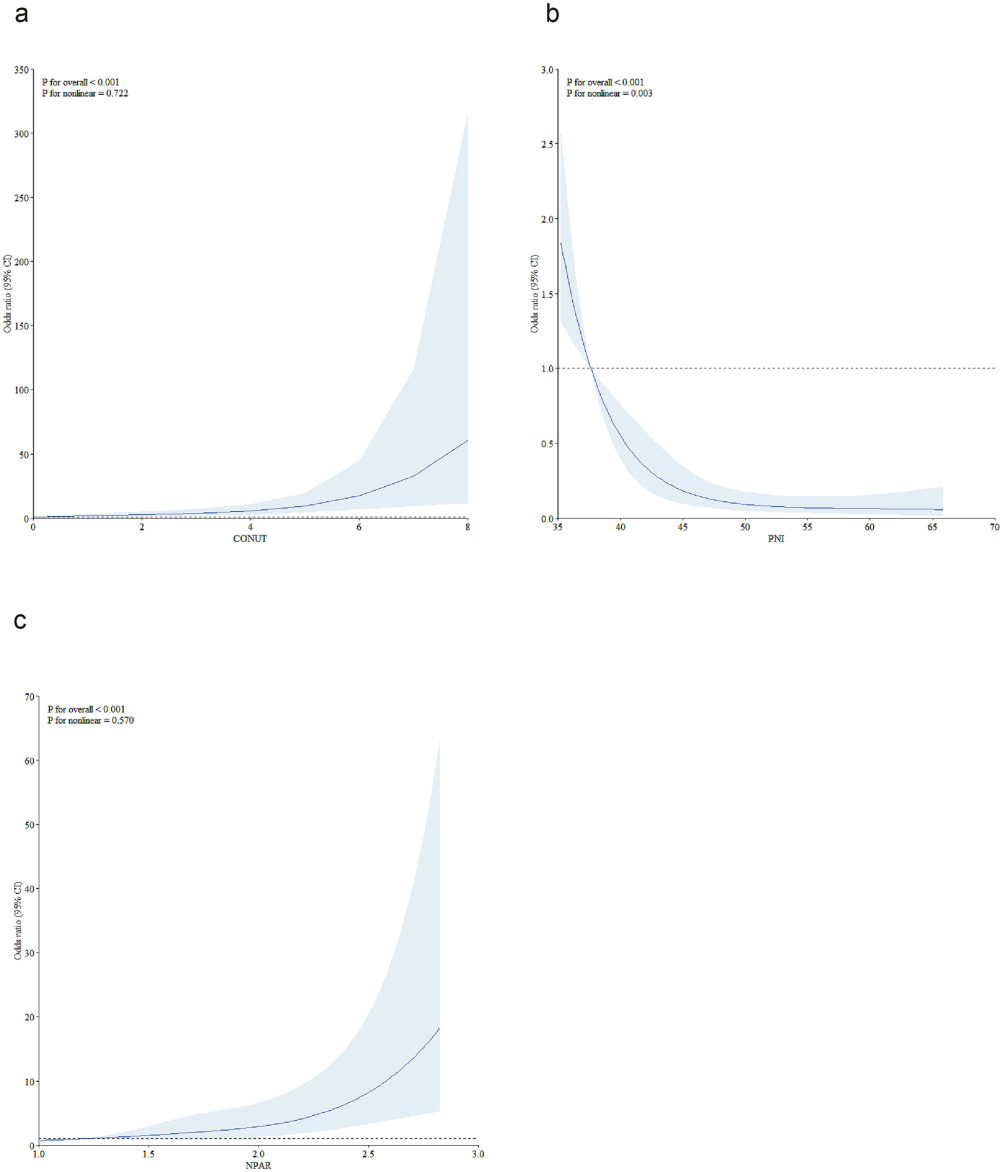
Restricted cubic spline analyses were performed to test nonlinear associations. To model the relationships of CONUT **(a)**, PNI **(b)**, and NPAR **(c)** with the 3-month risk of unfavorable outcomes in AIS patients treated with reperfusion therapy, restricted cubic spline models with 5 knots (at the 5th, 27.5th, 50th, 72.5th, and 95th percentiles) were constructed, adjusting for covariates from Model 3 in Table 3. In the plots, the solid line represents the hazard ratio, while the blue lines denote the 95% confidence intervals. CONUT, controlling nutritional status score; PNI, prognostic nutritional index; NPAR, neutrophil percentage-to-albumin ratio; AIS, acute ischemic stroke.

We further conducted RCS analyses to explore the potential nonlinear association between baseline malnutritional risk score and short-term prognosis. As shown in Figure 2, RCS analyses revealed no nonlinear associations between the CONUT/NPAR indices and poor outcomes (Nonlinear tests, for CONUT: *p* = 0.722; for NPAR: *p* = 0.570). For the PNI index, an inverse J-shaped association was observed: the risk decreased substantially at the initial stage, reaching a minimum at approximately 46.50, followed by a relative flattening of risk thereafter (P for nonlinearity = 0.003).

**Figure 3:**
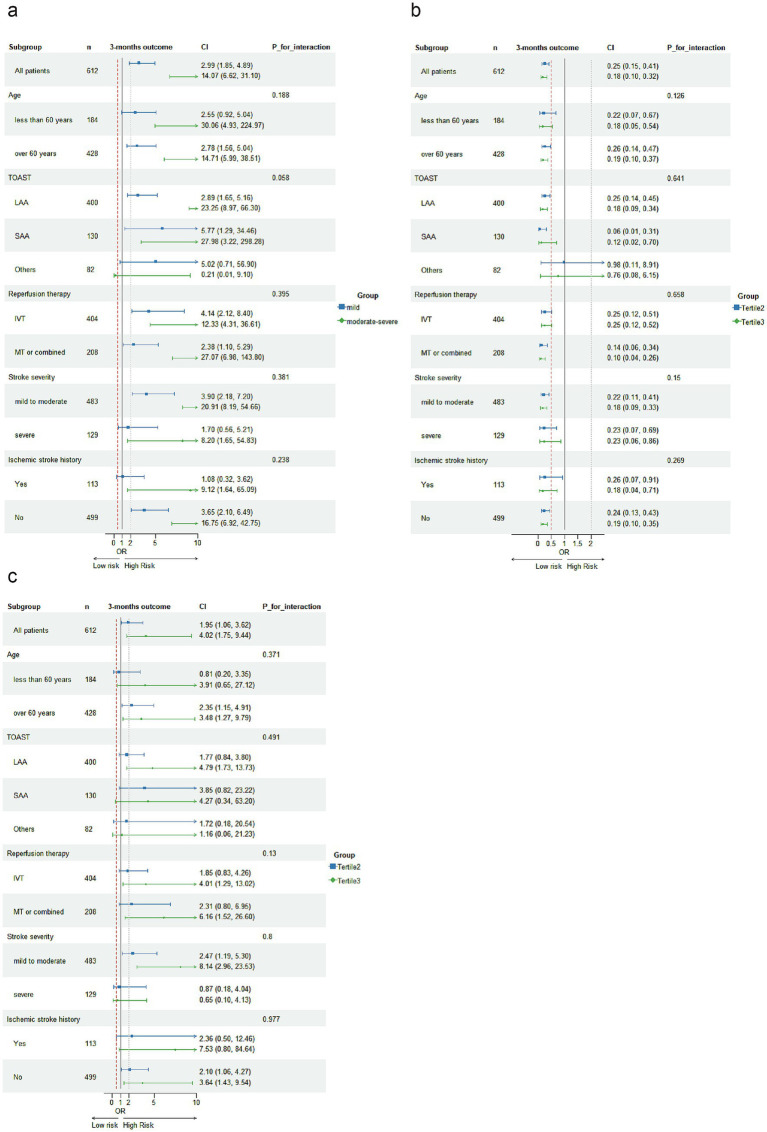
Subgroup analyses were conducted to investigate the association of malnutritional indices with 3-month unfavorable outcomes following acute ischemic stroke. Logistic regression analysis adjusted for covariates from Model 3 in Table 3 were used to examine the associations across different subgroups, with malnutrition indices categorized as: COUNT [absent risk, mild risk, moderate to severe risk, **(a)**], PNI tertiles **(b)**, and NPAR tertiles **(c)**.

In subgroup analysis, malnutrition defined by the COUNT scoring system and PNI tertiles classification remained significantly associated with poor prognosis in AIS patients, except in the subgroup of patients with other stroke etiologies. Malnutrition defined by NPAR tertiles showed no significant associations with poor prognosis in AIS patients across the following subgroups: those less than 60 years old, small-artery occlusion lacunar (SAA) etiology of stroke, other etiology of stroke, severe stroke, and non-first-ever stroke (Figure 3). No significant interactions were observed between the three malnutrition scores and variables including age, TOAST subtype, type of reperfusion therapy, stroke severity, and history of ischemic stroke in relation to poor prognosis among AIS patients with reperfusion therapy (*P* for interaction >0.05).

### 3.3 Incremental prognostic value of three malnutritional index for 3-month poor outcome

**Figure 4:**
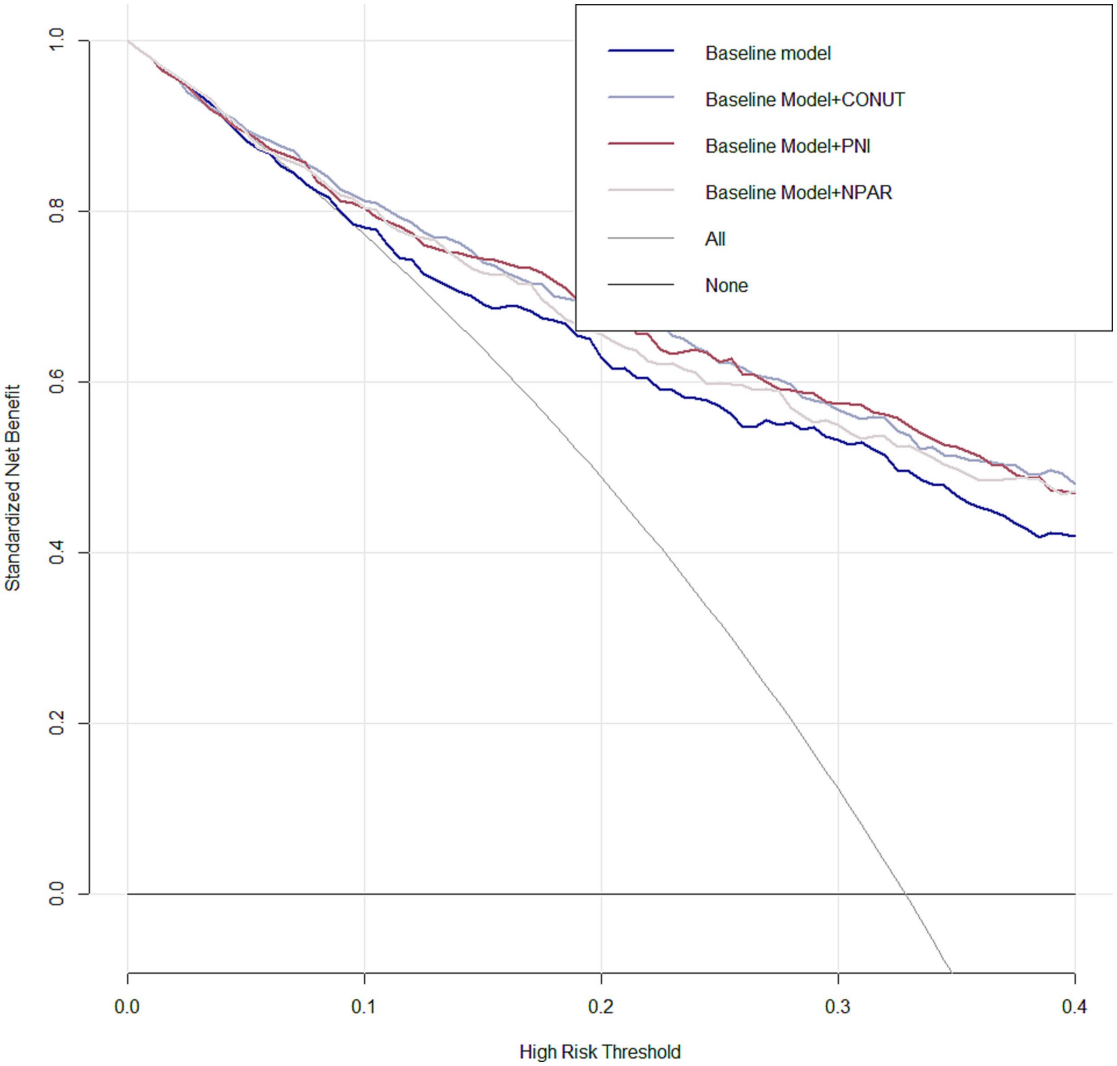
Decision curve analysis (DCA) was performed for Model 3 integrating PNI, CONUT, and NPAR features, compared with Model 3 without malnutritional indices. The deep red line representing the model integrating PNI, the sliver line representing the model integrating CONUT and the lavender line representing the model integrating NPAR.

By calculating the net reclassification improvement (NRI) and integrated discrimination improvement (IDI) indices, we demonstrated the incremental value of malnutrition indices in predicting 3-month unfavorable outcomes among AIS patients when combined with the traditional risk factors included in Model 3. This was evidenced by the positive NRI and IDI coefficients across all models (Table 4). When compared to NPAR, PNI and CONUT Score were similarly more effective in predicting unfavorable prognosis among AIS patients undergoing reperfusion therapy, as their IDI and NRI values were higher than those of NPAR. Moreover, decision curve analysis for the four models in predicting 3-month poor prognosis risk in AIS patients receiving IVT or/and EVT patients with AIS at 3 months is presented in Figure 4. The DCA demonstrates that incorporating PNI, CONUT, or NPAR features to predict poor prognosis provides greater net benefit compared to using only the clinical features from Model 3.

**Table 4:** Reclassification statistics (95% CI) and integrated discrimination improvement to predict 3-month poor prognosis after the addition of three malnutritional indices.

Model	C-index	cNRI	*p*-value	IDI	*p*-value
Model3^†^	0.814 (0.777–0.851)	Reference		Reference	
Model3 + PNI	0.850 (0.817–0.883)	0.579 (0.418–0.739)	**<0.001**	0.072 (0.049–0.095)	**<0.001**
Model3 + CONUT	0.851 (0.819–0.883)	0.631 (0.470–0.792)	**<0.001**	0.067 (0.044–0.089)	**<0.001**
Model3 + NPAR	0.837 (0.803–0.871)	0.416 (0.252–0.581)	**<0.001**	0.039 (0.022–0.056)	**<0.001**

†Model 3, adjusted for age, history of ischemic stroke, history of diabetes mellitus, systolic blood pressure, stroke etiology, the level of carotid atherosclerosis, white blood cell, Hemoglobin, triglyceride, fasting blood sugar, international normalized ratio, NIHSS at admission, treating-type of reperfusion therapy. CI, confidence interval; cNRI, continuous reclassification improvement; IDI, integrated discrimination improvement; CONUT, controlling nutritional status score; PNI, prognostic nutritional index; NPAR, neutrophil percentage-to-albumin ratio. Bold font values indicate statistically significant differences.

Additionally, the ROC curve demonstrated the predictive performance of CONUT (AUC: 0.733), PNI (AUC: 0.738) and NPAR (AUC: 0.711) for 3-month unfavorable outcome was close to good (AUC > 0.7, Supplementary Figure 1).

### 3.4 Mediation analyses

Furthermore, parallel mediation analyses were conducted to assess the potential mediation effects of END on the associations of malnutrition with 3-months unfavorable outcome risk in AIS undergoing reperfusion therapy. END had no significant mediated effects on the associations of CONUT, PNI, and NPAR with 3-months unfavorable outcome risk (all *p* < 0.05, Supplementary Figure 2).

## 4 Discussion

In the present study, we demonstrated that high CONUT and NPAR levels, along with low PNI levels, were independently associated with an increased risk of 3-month poor functional outcome in AIS patients undergoing reperfusion therapy (IVT/EVT alone or IVT bridged with EVT). The current study employed multiple statistical analyses to demonstrate that immune-nutritional assessment tools based on blood test indices are promising and cost-effective biomarkers, which can be used to predict short-term prognosis after reperfusion therapy. According to our knowledge, this is the first study with a relatively large samples size to focus on the prognostic value of diverse albumin-based malnutritional markers among the hyper-acute stage stroke patients no matter they undergoing IVT/EVT alone or EVT bridged with IVT.

Two critical risk factors that contribute to adverse outcome in patients with ischemic stroke are inflammation and malnutrition (32, 33). Emerging evidence supports that objective inflammatory-nutritional scores have been incorporated into current clinical decision algorithms for prognostic risk stratification in ischemic stroke patients. Recent management guidelines for ischemic stroke advocate that nutritional assessment should be performed at baseline for all stroke patients (34), and early recognition of individual malnutrition would improve the outcome (35). Nonetheless, the growing burden of nutritional assessment has consistently been deemed challenging, primarily attributed to the time-sensitive characteristics of ischemic stroke progression. Moreover, the side effect including bleeding, ischemia reperfusion injury with edema, and angioedema after clot dissolving, and post-recanalization no-reflow phenomenon may arise after reperfusion therapy, which would pose more challenge on AIS early management (36–38). Hence, the easy estimation of immune-nutritional markers calculating form the blood test indices could be suitable and feasible options in acute stroke settings.

Prior investigations into this topic have demonstrated that objective malnutrition correlates with short-term clinical prognosis, as assessed using the CONUT, PNI, and NPAR scores (5, 7, 15, 21, 39, 40). The above-mentioned researches primarily have established the prognostic significance of the CONUT, PNI or NPAR in stroke populations undergoing IVT or EVT alone, its association with clinical outcomes in general reperfusion therapy-treated patients remains unclear. In the context of prompt reperfusion strategy involving IVT/EVT alone, or EVT combined with IVT (bridged therapy) remains the first-line treatment in ischemic stroke intensive practice (41), our main findings answer the clinical concerns as follows: (1) CONUT, PNI and NPAR score could be used to predict 3-months poor prognosis risk after reperfusion therapy in AIS patients; (2) compared to NPAR, the predictive ability of CONUT and PNI scores were seem to be more reliable since the NRI and IDI value of the two indices were higher than NPAR. Additionally, the potential influence of nonlinear dose–response relationship of PNI and 3-months worse outcome in AIS patients undergoing reperfusion therapy should be considered. Sensitivity analyses conducted on participants with age > 60 years old, with stroke subtype of LAA, with stroke subtype of SAA, with mild to moderate stroke severity (NIHSS at admission less than 20), with IVT alone, with EVT alone or combined with IVT, with first episode ischemic stroke, further validated the relationships of CONUT and PNI among these individuals, confirming the robustness of our results.

Although the mechanisms through such composite albumin-based malnutritional markers influence clinical outcomes in ischemic stroke patients following reperfusion therapy remain incompletely elucidated, several potential pathophysiological pathways have been hypothesized. Post-ischemic inflammation exerts a pivotal role in the pathophysiological response to cerebral ischemia–reperfusion injury among AIS patients after reperfusion therapy (42). In the acute phase, immune-inflammatory cells such as polymorphonuclear neutrophils and lymphocytes, alongside lymphocyte activation, may initiate the release of pro-inflammatory cytokines and cytotoxic mediators, thereby further mediating secondary neuronal damage (43). Conversely, accumulating evidence suggests that lymphocytes also play indispensable roles in chronic tissue repair and remodeling processes (44). A Chinese cohort study demonstrated that elevated admission lymphocyte counts were associated with lower risks of mortality, stroke recurrence, and unfavorable neurological prognosis (45). Serum albumin has been demonstrated neuroprotective attributes, including its capacity to inhibit erythrocyte aggregation (46) and the role as a major antioxidant (47). Decreased serum albumin levels have been significantly associated with adverse clinical outcomes in patients undergoing EVT (48). The association between total cholesterol (TC) and stroke remains equivocal. Low TC level in stroke patients may act as a double-edged sword: while potentially reducing atherosclerosis risk, they concurrently increase susceptibility to hemorrhagic transformation by promoting endothelial injury through necrosis of smooth muscle cells in the arterial media (49, 50), which the risk may more apparent in patients with reperfusion therapy. Among the reported nutritional biomarkers, the CONUT score and PNI score could better reflect the underlying nutritional and inflammatory mechanisms in subjects as well as could serve as more appropriate biomarkers for predicting short-term prognosis after reperfusion therapy in AIS.

Despite the availability of multiple malnutrition screening tools, there remains no consensus on the optimal objective marker for acute ischemic stroke (AIS) patients undergoing reperfusion therapy. In light of the aforementioned findings, we recommend that the CONUT score or PNI score could be employed, owing to their incremental value in risk forecasting. It may be beneficial for these patients to participate in targeted secondary prevention programs and receive nutritional intervention, with the aim of improving their prognosis.

The current investigation is subject to several inherent limitations. First, as a single-center retrospective study conducted in a Chinese medical institution, it may be susceptible to potential selection bias. Secondly, given the retrospective design, dynamic malnutritional markers were not evaluated, which precluded analysis of the impact of longitudinal changes in these nutritional indices on prognosis throughout the follow-up period. However, we performed a mediation analysis to demonstrated that exposure to malnutritional status increased 3-month poor prognosis risk, which was not partly mediated by the incidence of END in patients treated with IVT or EVT. Third, akin to all observational studies, this study may be influenced by unmeasured or uncontrolled confounding variables, even after adjusting for acknowledged potential confounders.

## 5 Conclusion

Our findings demonstrate that the imbalance of inflammation and nutritional status in AIS patients undergoing reperfusion therapy is associated with a heightened risk of short-term poor prognosis. The CONUT and PNI scores may serve as valuable malnutritional biomarkers, not only for predicting clinical outcomes but also for guiding prognostic optimization in post-IVT and/or EVT patients by identifying those who might benefit from acute-stage nutritional intervention. Further studies are warranted to evaluate the efficacy of nutritional management strategies based on these two indicators in AIS populations.

## Data Availability

The raw data supporting the conclusions of this article will be made available by the authors, without undue reservation.
