# Supplementary material for: Prognostic value of albumin-based malnutritional indices on short-term outcome in acute ischemic stroke patients undergoing reperfusion therapy
Source: Front Nutr. 2025 Aug 4;12:1659446. doi: 10.3389/fnut.2025.1659446 (PMC12358433; doi:10.3389/fnut.2025.1659446)
Supplement: Supplementary file 1 [file Data_Sheet_1.docx]

Supplementary Material

# Supplementary Figures


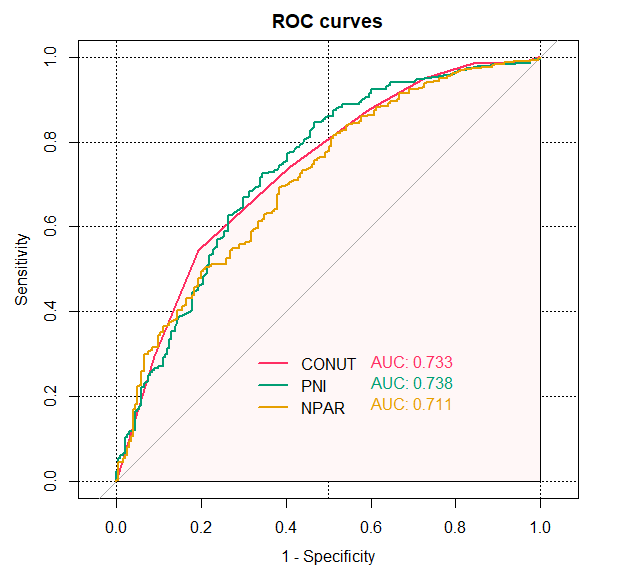


**Supplementary Figure 1.** ROC curve showing the predictive ability of CONUT score, PNI and NPAR for 3-month unfavorable outcomes. Abbreviations: ROC, receiver operating characteristics; CONUT, controlling nutritional status; PNI, prognostic nutritional index; NPAR, neutrophil percentage-to-albumin ratio.


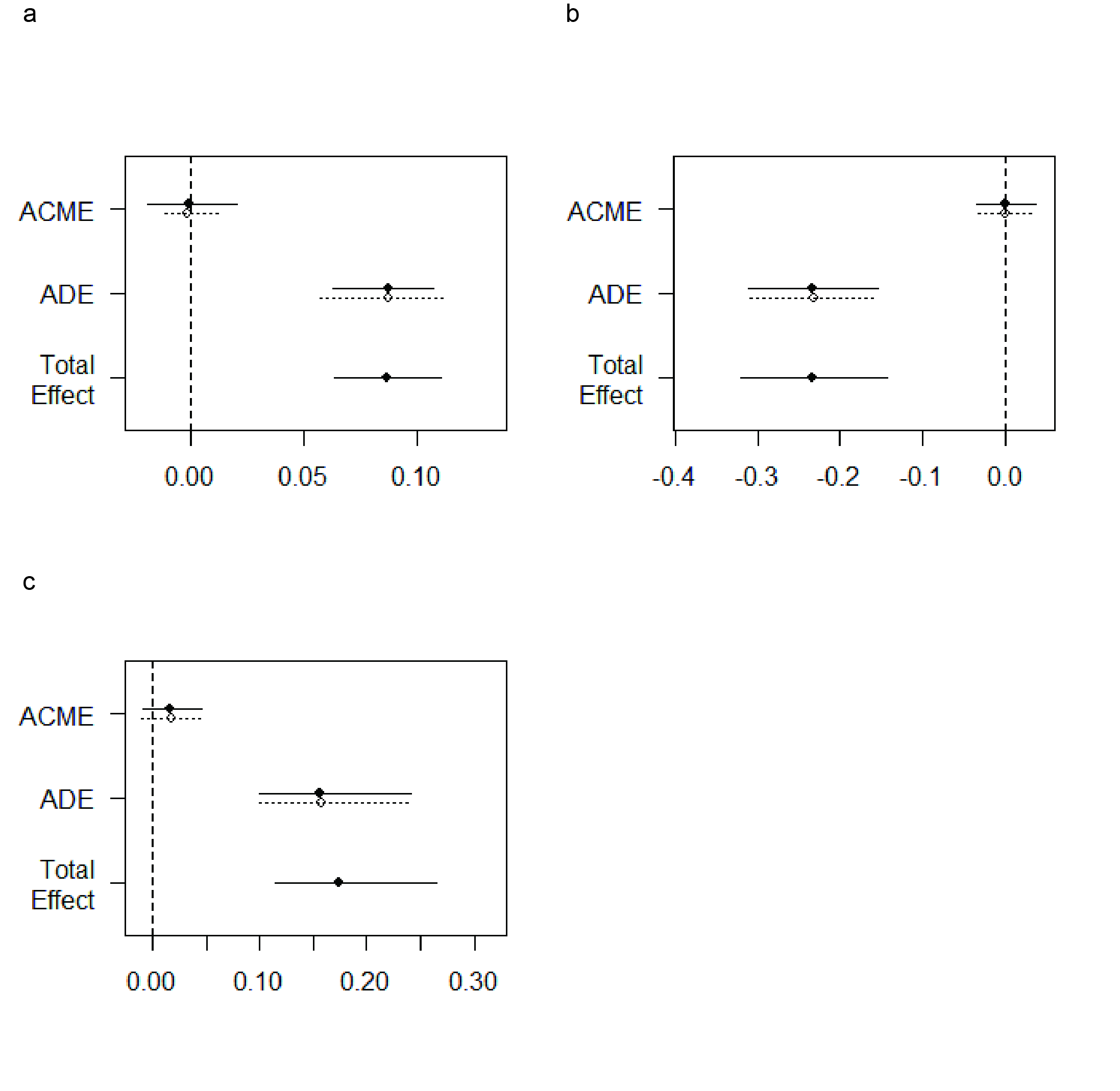


Supplementary Figure 2. Estimated proportion of the association between malnutritional indices and 3-months unfavorable functional outcome by early neurological deterioration CONUT(a), PNI (b), and NPAR (C). Models were adjusted for age, history of ischemic stroke, history of diabetes mellitus, systolic blood pressure, stroke etiology, the level of carotid atherosclerosis, white blood cell, Hemoglobin, triglyceride, fasting blood sugar, international normalized ratio, NIHSS at admission, treating-type of reperfusion therapy. ACME, the estimate of the indirect effect; ADE, the estimate of the direct effect.
